# First-line treatment patterns and real-world survival outcomes in PD-L1-negative locally recurrent inoperable or metastatic triple-negative breast cancer in the United States

**DOI:** 10.1007/s10549-026-08040-1

**Published:** 2026-08-05

**Authors:** Tiffany A. Traina, Elizabeth Serra, Jerome Bedard, Mohira Levesque-Leroux, Patrick Gagnon-Sanschagrin, Annie Guérin, Silke Guenther, George Joseph, Victoria Guan, Jonathan Salcedo

**Affiliations:** 1 Department of Medicine, Evelyn H. Lauder Breast Center, Memorial Sloan Kettering Cancer Center, New York, NY USA; 2 Weill Cornell Medicine, New York City, NY USA; 3 Analysis Group ULC, Montréal, QC Canada; 4 BioNTech SE, Mainz, Germany; 5 BioNTech US, Inc., Cambridge, MA USA

**Keywords:** Breast cancer, Triple-negative breast cancer, Treatment patterns, Overall survival, Progression

## Abstract

**Purpose:**

Contemporary real-world data on treatment patterns and clinical outcomes in patients with programmed death ligand 1 (PD-L1)-negative locally recurrent inoperable or metastatic triple-negative breast cancer (lr/mTNBC) are sparse. We describe first-line (1L) systemic therapies and real-world survival outcomes in this population in the United States (US).

**Methods:**

Adults with PD-L1-negative lr/mTNBC initiating 1L systemic treatment in the US were identified using the Komodo Research Data (KRD+; 1/1/2016-12/31/2023). Treatment patterns, including treatment durations and sequences from 1L through three lines of therapy, real-world overall survival (rwOS) and progression-free survival (rwPFS) from 1L, were analyzed. rwOS and rwPFS were summarized using Kaplan-Meier methods in subgroups of patients with ≥ 18 months and ≥ 6 months of potential follow-up, respectively.

**Results:**

Overall, 929 patients were included (median age 59.0 years, 60.1% White, 26.4% Black or African American, 64.9% commercially insured). Most common 1L treatments included cyclophosphamide + doxorubicin-based regimens (28.1%), capecitabine (12.7%), and paclitaxel (10.4%); 44.5% were observed to receive a second-line treatment, and 16.0% a third-line treatment. Treatment sequences were heterogeneous, with 329 unique sequences captured. Median rwOS and rwPFS (95% confidence interval) were 12.2 (10.7, 14.4) months and 4.7 (4.5, 5.2) months, respectively.

**Conclusion:**

In this large real-world US cohort of patients with PD-L1-negative lr/mTNBC, 1L treatment regimens were highly heterogeneous and often diverged from guideline recommendations. Despite treatment, survival outcomes remain poor within this patient population. These findings highlight the need for more effective therapeutic options for this population.

## Introduction

Triple-negative breast cancer (TNBC), which accounts for approximately 15–20% of incident breast cancer (BC), is a highly aggressive subtype characterized by a lack of estrogen receptors, progesterone receptors, and human epidermal growth factor receptor 2 (HER2) expression or amplification [1]. TNBCs are biologically heterogeneous tumors with several distinct molecular subtypes [1–3]. They exhibit high proliferative activity, an aggressive clinical phenotype, and a high risk for distant metastases to the brain, lungs, liver, and bones [1, 2, 4]. Accordingly, patients with TNBC face a poor prognosis, with a higher likelihood of distant recurrence relative to other BC subtypes [5]. Globally, among patients with locally recurrent inoperable or metastatic TNBC (lr/mTNBC), the median overall survival (OS) ranges from approximately 12 to 18 months [6–10] and the 5-year survival rates range from approximately 10% to 15% [8, 11].

Treatment options for patients with lr/mTNBC have been an important focus of research given its aggressive nature and poor prognosis. However, until recently, therapeutic options for patients with lr/mTNBC had remained largely unchanged for over a decade [9]. Since 2018, several novel targeted therapies have emerged for select subpopulations, starting with the two poly (ADP-ribose) polymerase inhibitors, olaparib and talazoparib, for those with the *BReast CAncer gene 1* (*BRCA1)* or *BReast CAncer gene 2* (*BRCA2)* pathogenic variant [12]. In 2020, the immune checkpoint inhibitor (ICI) pembrolizumab was approved for use in combination with chemotherapy for patients with programmed death ligand 1 (PD-L1)-positive lr/mTNBC after demonstrating a significant improvement in OS compared with chemotherapy alone in the KEYNOTE-355 clinical trial [12, 13]. In contrast, patients with PD-L1-negative lr/mTNBC continue to have limited treatment options outside of clinical trials, with practice guidelines recommending single-agent cytotoxic chemotherapy (e.g., capecitabine, paclitaxel) as the preferred first-line (1L) treatment approach [9]. Numerous clinical trials are underway to investigate novel genomically-targeted therapies for patients with PD-L1-negative tumors who remain ineligible for ICI-based regimens [12], which may inform current treatment strategies and improve the 1L standard of care for this population. Notably, results from the ASCENT-03 and TROPION-Breast02 trials reported promising survival benefits with sacituzumab govitecan-hziy and datopotamab deruxtecan (both Trop-2-directed antibodies) as a 1L treatment option in patients with PD-L1-negative lr/mTNBC compared to chemotherapy [14, 15].

Although many clinical trials are currently exploring new therapeutic options for this population, contemporary real-world data on treatment patterns and clinical outcomes among patients with PD-L1-negative lr/mTNBC remain limited. Characterizing the real-world outcomes among patients with PD-L1-negative lr/mTNBC will be critical to contextualize results from the ongoing clinical trials. Furthermore, a better understanding of how these patients are treated in routine clinical practice is essential to support educated treatment decision-making for this high-risk population. To address this gap in knowledge, this retrospective cohort study describes the treatment patterns and real-world clinical outcomes of patients with PD-L1-negative lr/mTNBC in the United States (US).

## Methods

### Data source

The Komodo Research Data (KRD+) database, covering the period from January 1, 2016, to December 31, 2023 (end of data availability), was used for this study. KRD+ is a US healthcare claims data source containing patient-level demographic information including age, sex, race/ethnicity, and geographical residence. Administrative claims data elements within KRD+ included, but were not limited to, inpatient (IP) and outpatient (OP) diagnosis and procedure codes, drug prescriptions, provider information, and payer type. This study was considered exempt research under 45 CFR § 46.104(d)(4) because it involved only the secondary use of data that were de-identified in compliance with the Health Insurance Portability and Accountability Act, specifically, 45 CFR § 164.514.

### Study design and population

A retrospective, longitudinal, cohort study was used to describe treatment patterns, real-world OS (rwOS), and real-world progression-free survival (rwPFS) for patients with PD-L1-negative lr/mTNBC. The index date was defined as the initiation of the first systemic treatment for lr/mTNBC (Supplemental Fig. 1). Patients were required to have continuous health plan enrollment from ≥ 12 months prior to the first observed BC diagnosis to the earliest of (i) end of continuous health plan enrollment, (ii) end of data availability, or (iii) death (Supplemental Fig. 1).

Patients were included if they met the following eligibility criteria: (i) had ≥ 2 International Classification of Diseases, 10th Revision, Clinical Modification (ICD-10-CM) codes for BC (C50.x) on distinct dates ≥ 30 days apart, (ii) had inoperable BC identified using a claims-based algorithm based on medical expert input, characterized by the presence of a biopsy followed by the initiation of a systemic treatment for BC within 3 months, and no BC surgery within 12 months of the biopsy, (iii) had no hormone receptor (HR), HER2 or PD-(L)1-specific treatments in the lr/mTNBC setting, and (iv) were ≥ 18 years of age at initiation of first systemic treatment for lr/mTNBC. Additionally, patients were considered to have lrBC if there was evidence of early-stage BC and the biopsy during the inoperable BC period occurred more than 9 months after the first BC diagnosis. Patients were classified as having *de novo* mBC if the biopsy occurred within 9 months of first BC diagnosis, there was evidence of a distant metastatic site, and there was no evidence of prior early-stage disease. Because clinical biomarker information is not directly available in KRD+, triple-negative and PD-L1-negative status were approximated using the absence of HR-, HER2-, and PD-(L)1-targeted therapies (i.e., pembrolizumab and atezolizumab) in the lr/mTNBC setting. Patients with indicators of BRCA1/2 pathogenic variants, including diagnosis codes for genetic susceptibility to malignant neoplasm of breast or receipt of BRCA1/2-specific treatments (i.e., olaparib or talazoparib) at any time, were excluded.

### Study measures, outcomes, and analyses

Patient characteristics were described as of the index date, and included age, sex, race/ethnicity, payer type, US census region of residence, and calendar year of index date. Clinical characteristics included disease stage (lr TNBC and *de novo* mTNBC) at index date, the National Cancer Institute (NCI) comorbidity index score, and individual NCI comorbidities assessed in the 12 months prior to the index date.

Lines of therapy (LOTs) from the index date to the earliest of (i) end of continuous health plan enrollment, (ii) end of data availability, or (iii) death were identified using a claims-based algorithm based on observed changes in treatment regimens, such as treatment discontinuation and treatment switch. Treatment patterns, including treatment duration and sequences, of up to three consecutive LOTs per patient were described.

rwOS was assessed from the index date until death or end of data availability (censor) among the subgroup of patients with an index date ≥ 18 months before the end of data availability (i.e., ≥ 18 months of potential follow-up before censoring at the end of data availability, unless death occurred earlier). Mortality information available in KRD+ was supplemented using an algorithm developed with medical expert input and informed by previously published literature [16]. Patients without a date of death (reported in KRD+ or identified through the algorithm) were considered alive at the end of data availability.

rwPFS was assessed from the index date until the first observed real-world indicator of progression or censor date among the subgroup of patients with an index date ≥ 6 months before the end of data availability (i.e., ≥ 6 months of potential follow-up). The algorithm used to identify indicators of progression was developed with medical expert input. Patients without any real-world indicator of progression were considered not to have progressed and were censored at the end of continuous health plan enrollment or end of data availability, whichever occurred first. Full methodological details are reported in the Supplementary Methods.

Descriptive statistics were reported for the measures described above. Means, standard deviations, and medians were reported for continuous variables and counts and percentages were reported for categorical variables. Kaplan-Meier analyses were used to estimate rwOS and rwPFS. A rwOS sensitivity analysis was conducted among patients with an index date on or after March 2019, corresponding to the approval of the first PD-(L)1-targeted therapy in this setting. All analyses were conducted using SAS^®^ Enterprise Guide 8.3 (SAS Institute, Inc., 100 SAS Campus Dr Cary, NC 27513, USA).

## Results

### Patient characteristics

**Fig. 1:**
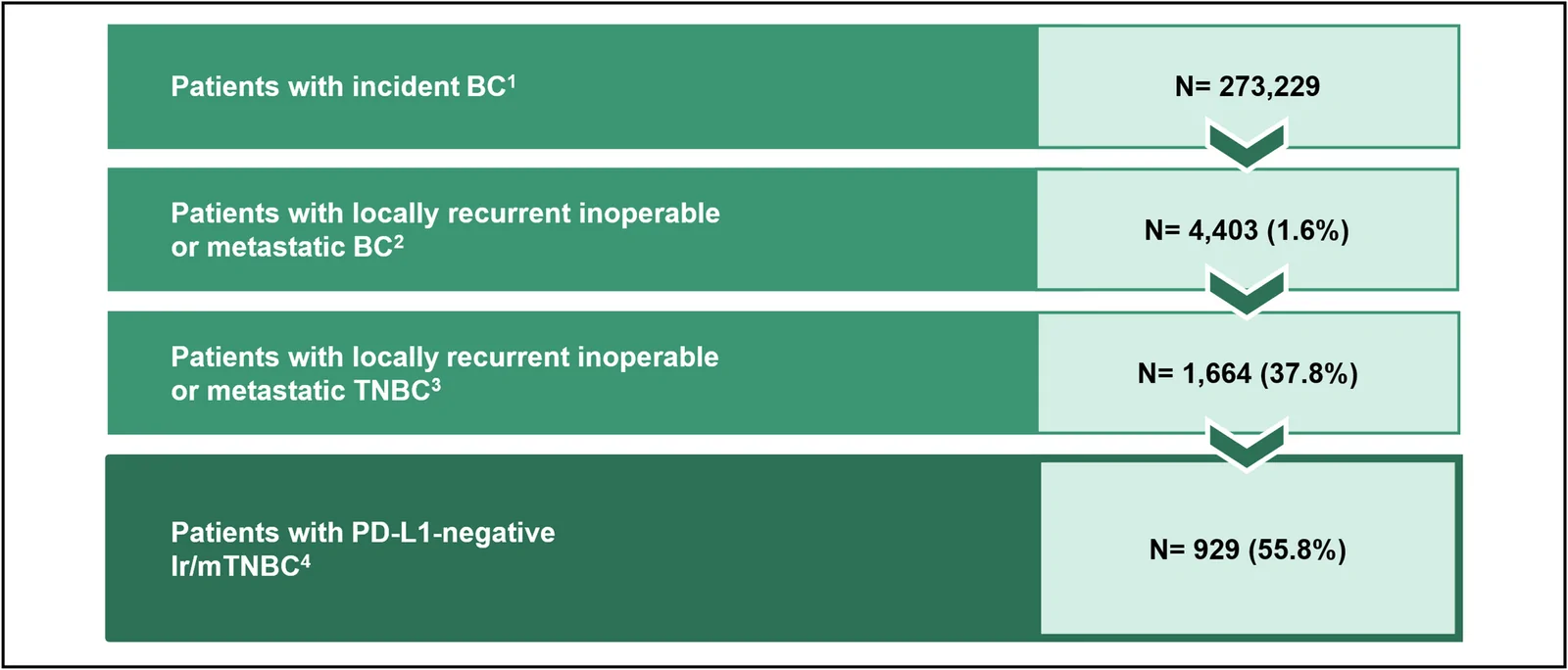
Sample selection flowchart. Abbreviations: BC: breast cancer; BRCA: BReast CAncer gene; HER: human epidermal growth factor receptor; HR: hormone receptor; ICD-10-CM: International Classification of Diseases, 10th Edition, Clinical Modification; lr/mTNBC: locally recurrent inoperable or metastatic triple-negative breast cancer; PD-L1: Programmed Death-Ligand 1. Notes: 1. Patients with incident BC were required to have ≥ 2 claims for BC (ICD-10-CM C50.x) ≥ 30 days apart, ≥ 12 months of continuous health plan enrollment prior to their first BC diagnosis, and no indicator of prior cancer (defined as diagnoses of other primary malignant neoplasms [ICD-10-CM C00-C96, except C43-C44 and C4A.x, C50.x, C77-C79, and C7B.x], personal history of malignant neoplasm [Z85.x] and codes for chemotherapy and for general radiotherapy). 2. Patients with lr/mBC were required to have a biopsy with no breast surgery ≤ 12 months after, and a systemic treatment ≤ 3 months after biopsy. 3. Patients with lr/mTNBC were required to have no HR- and HER-specific treatments in recurrent/metastatic setting. 4. Patients with PD-L1-negative lr/mTNBC were required to have no PD-(L)1-specific treatments in recurrent/metastatic setting. Patients with indicators of BRCA1/2 mutations, including diagnosis for genetic susceptibility to malignant neoplasm of breast or receiving BRCA1/2-specific treatments (olaparib or talazoparib) at any time, were excluded from the sample

A total of 929 patients with PD-L1-negative lr/mTNBC who initiated systemic treatment for BC were included (Fig. 1). Patients had a median age of 59.0 years, nearly all were female (98.8%), and most patients were commercially insured (64.9%). Among patients with known race and ethnicity, 60.1% were White, 26.4% were Black or African American, and 9.2% were Hispanic or Latino. Nearly two thirds were classified as having *de novo* mTNBC (65.4%). The three most prevalent baseline comorbidities were liver disease (26.8%), chronic obstructive pulmonary disease (22.7%), and diabetes (22.0%). Full patient characteristics are reported in Table 1 and characteristics of the subgroup of patients included in rwOS and rwPFS analyses are included in Supplemental Table 1.

**Table 1:** Patient characteristics

	Patients with PD-L1-negative lr/mTNBC
	N = 929
**Demographics at index date**	
**Age (years), mean ± SD [median]**	**58.9 ± 12.6 [59.0]**
18–24, N (%)	1 (0.1%)
25–34, N (%)	25 (2.7%)
35–44, N (%)	93 (10.0%)
45–54, N (%)	216 (23.3%)
55–64, N (%)	317 (34.1%)
65–74, N (%)	167 (18.0%)
75–84, N (%)	89 (9.6%)
85 or older, N (%)	21 (2.3%)
**Sex, N (%)**	
Sex known	927 (99.8%)
Female	916 (98.8%)
Male	11 (1.2%)
Sex unknown	2 (0.2%)
**Race & ethnicity, N (%)**	
Race & ethnicity known	644 (69.3%)
White	387 (60.1%)
Black or African American	170 (26.4%)
Hispanic or Latino	59 (9.2%)
Asian or Pacific Islander	18 (2.8%)
Other	10 (1.6%)
Race & ethnicity unknown	285 (30.7%)
**Payer type, N (%)**	
Commercial	603 (64.9%)
Medicare	248 (26.7%)
Medicaid	78 (8.4%)
**Year of index date, N (%)**	
2017–2019	359 (38.6%)
2020	162 (17.4%)
2021	155 (16.7%)
2022	165 (17.8%)
2023	88 (9.5%)
**Region of residence, N (%)**	
South	367 (39.5%)
Northeast	225 (24.2%)
Midwest	220 (23.7%)
West	116 (12.5%)
Unknown	1 (0.1%)
**Clinical characteristics**	
**Follow-up duration**^1^ **(months), mean ± SD [median]**	**15.6 ± 14.5 [10.3]**
**Disease stage at index, N (%)**	
De-novo mTNBC	608 (65.4%)
Locally recurrent inoperable TNBC	321 (34.6%)
**NCI Comorbidity Index**^2^, **mean ± SD [median]**	**0.57 ± 0.60 [0.52]**
Liver disease, N (%)	249 (26.8%)
Chronic obstructive pulmonary disease, N (%)	211 (22.7%)
Diabetes, N (%)	204 (22.0%)
Peripheral vascular disease, N (%)	126 (13.6%)
Renal disease, N (%)	80 (8.6%)
Cerebrovascular disease, N (%)	75 (8.1%)
Congestive heart failure, N (%)	74 (8.0%)
Rheumatologic disease, N (%)	46 (5.0%)
Myocardial infarction, N (%)	27 (2.9%)
Hemiplegia or paraplegia, N (%)	18 (1.9%)
Dementia, N (%)	15 (1.6%)
Peptic ulcer disease, N (%)	11 (1.2%)
AIDS/HIV, N (%)	3 (0.3%)

Abbreviations: AIDS/HIV: acquired immunodeficiency syndrome/human immunodeficiency virus; lr/mTNBC: locally recurrent inoperable or metastatic triple-negative breast cancer; N: number; NCI: National Cancer Institute; PD-L1: Programmed Death-Ligand 1; SD: standard deviation

Notes: 1. Defined as the time from index date to the earliest of end of continuous health plan enrollment, end of data availability, or death. 2. Assessed during the 12-month period prior to the index date

### Treatment patterns

**Fig. 2:**
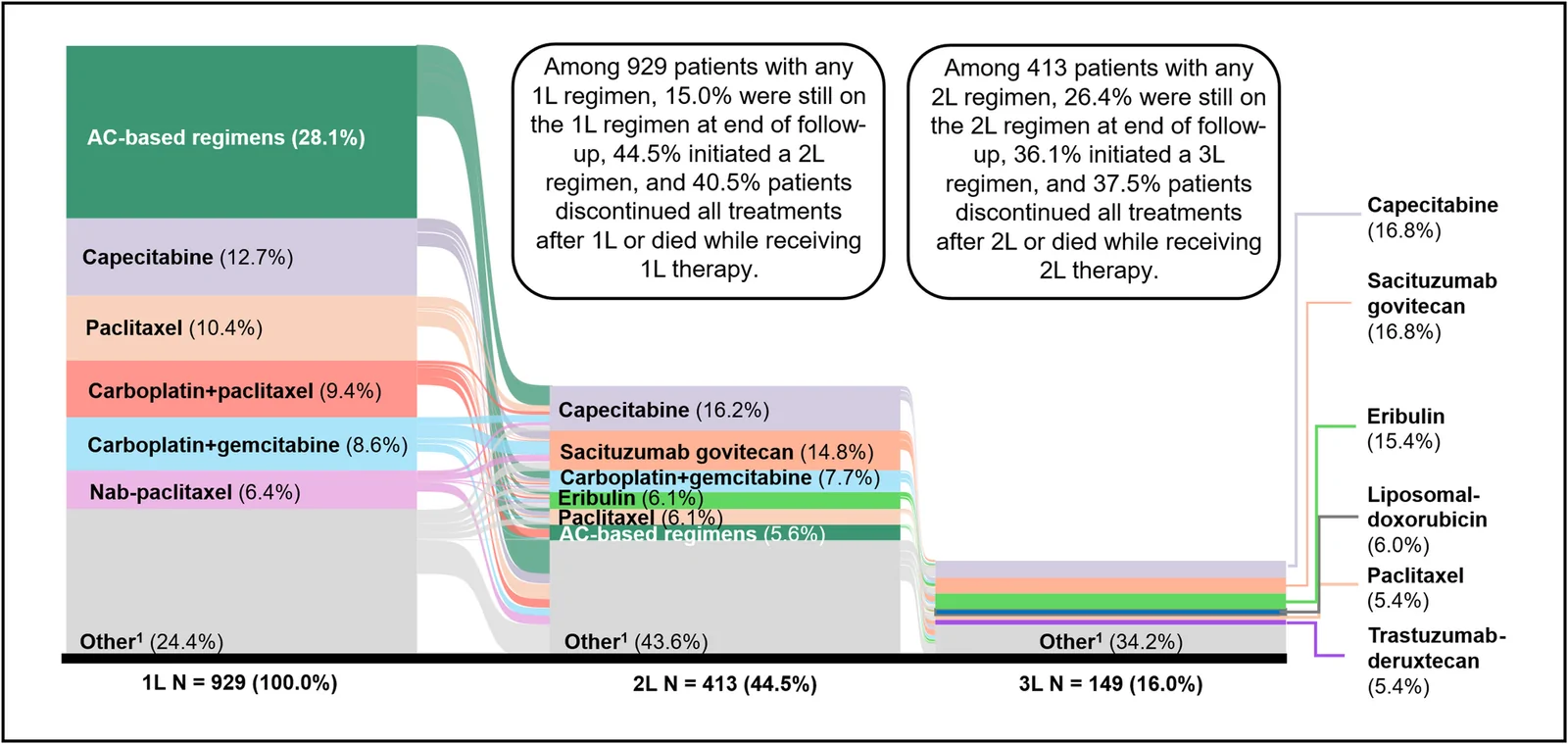
Treatment sequences from initiation of 1L treatment for PD-L1-negative lr/mTNBC. Abbreviations: 1L: first line; 2L: second line; 3L: third line; AC: cyclophosphamide + doxorubicin; lr/mTNBC: locally recurrent inoperable or metastatic triple-negative breast cancer; PD-L1: Programmed Death-Ligand 1. Note: 1. Regimens representing less than 5% of the sample are grouped and reported as “other”. The five most frequent regimens within the “other” category are 1L: carboplatin (3.6%), methotrexate (2.6%), cyclophosphamide + docetaxel (2.4%), fluorouracil (2.4%), and eribulin (1.6%); 2L: carboplatin + paclitaxel (4.1%), nab-paclitaxel (4.1%), liposomal doxorubicin (3.9%), gemcitabine (2.9%), and trastuzumab deruxtecan (2.7%); 3L: gemcitabine (4.7%), carboplatin + gemcitabine (4.0%), nab-paclitaxel (3.4%), ixabepilone (2.0%), and carboplatin (1.3%)

Treatment sequences for PD-L1-negative lr/mTNBC were highly heterogeneous, with 329 unique sequences observed among 929 patients (Fig. 2). In 1L, the most common regimens were cyclophosphamide + doxorubicin (AC)-based regimens (28.1%), followed by capecitabine (12.7%), paclitaxel (10.4%), carboplatin + paclitaxel (9.4%), and carboplatin + gemcitabine (8.6%). Fewer than half (44.5%) of the patients received any second-line (2L) regimen, most commonly capecitabine (16.2%), sacituzumab govitecan (14.8%), or carboplatin + gemcitabine (7.7%). Only 16.0% of patients received a third LOT, including capecitabine (16.8%), sacituzumab govitecan (16.8%), or eribulin (15.4%). Duration of LOTs were short, with a median of 3.3 months in 1L, 2.8 months in 2L, and 2.6 months in third line (3L). Overall, 40.5% of patients discontinued all systemic treatments after 1L or died while receiving 1L therapy.

### rwOS

**Fig. 3:**
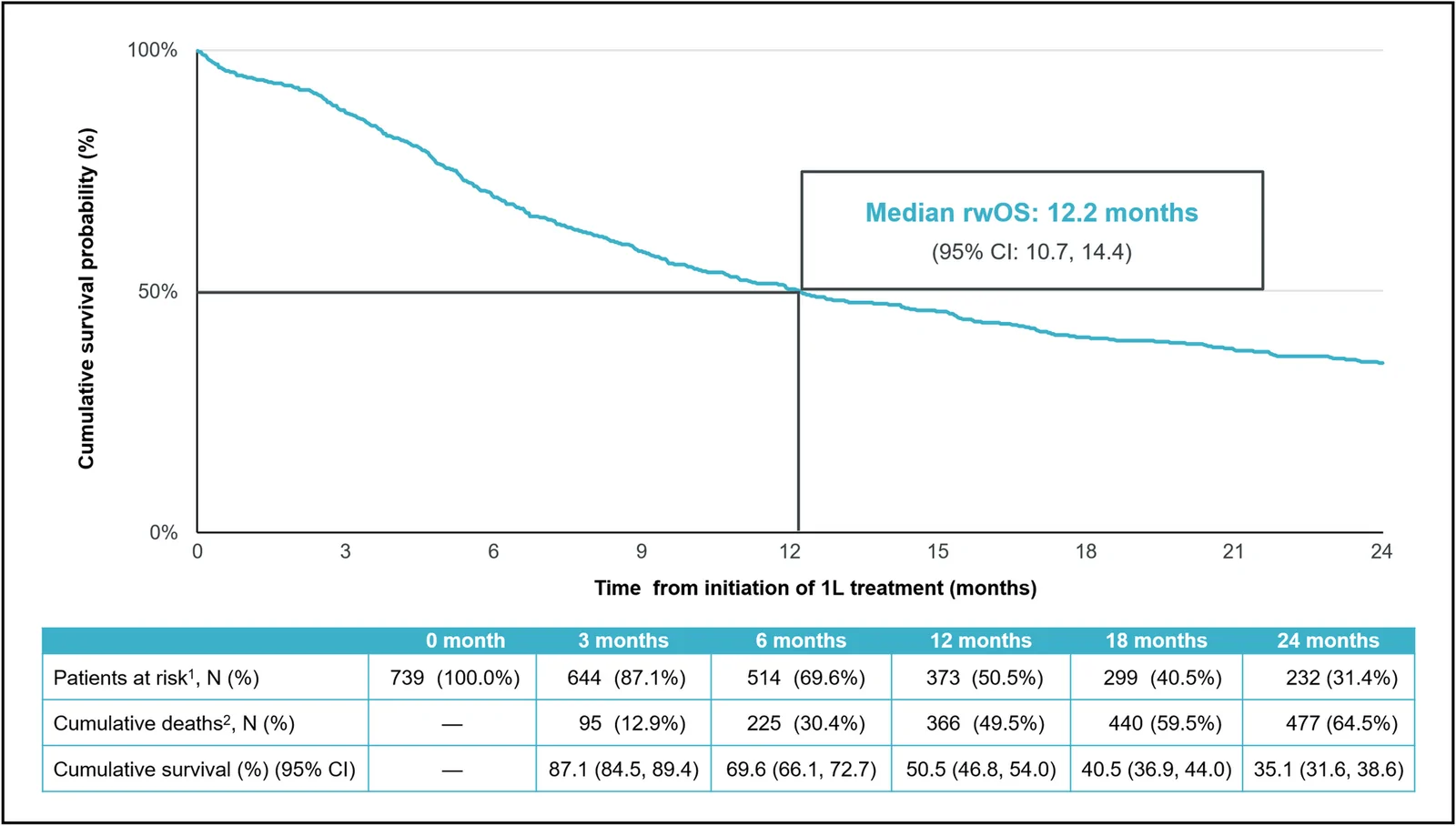
rwOS from initiation of 1L treatment for PD-L1-negative lr/mTNBC. Abbreviations: 1L: first line; CI: confidence interval; lr/mTNBC: locally recurrent inoperable or metastatic triple-negative breast cancer; PD-L1: Programmed Death-Ligand 1; rwOS: real-world overall survival. Notes: 1. Kaplan-Meier analyses were assessed among patients who initiated their 1L regimen for lr/mTNBC ≥ 18 months prior to the end of data availability. 2. Among 536 patients identified with a death date, 209 (39.0%) had a death date identified by the rwOS claims-based algorithm

Among 739 patients with sufficient follow-up to assess rwOS, 536 (72.5%) had died, and median rwOS was 12.2 (95% confidence interval [CI]: 10.7, 14.4) months (Fig. 3). rwOS rates at 3, 6, 12, and 18 months were 87.1%, 69.6%, 50.5%, and 40.5%, respectively. Results from the sensitivity analysis restricted to patients with an index date on or after March 2019 were consistent with those of the primary analysis (Supplemental Fig. 2).

### rwPFS

**Fig. 4:**
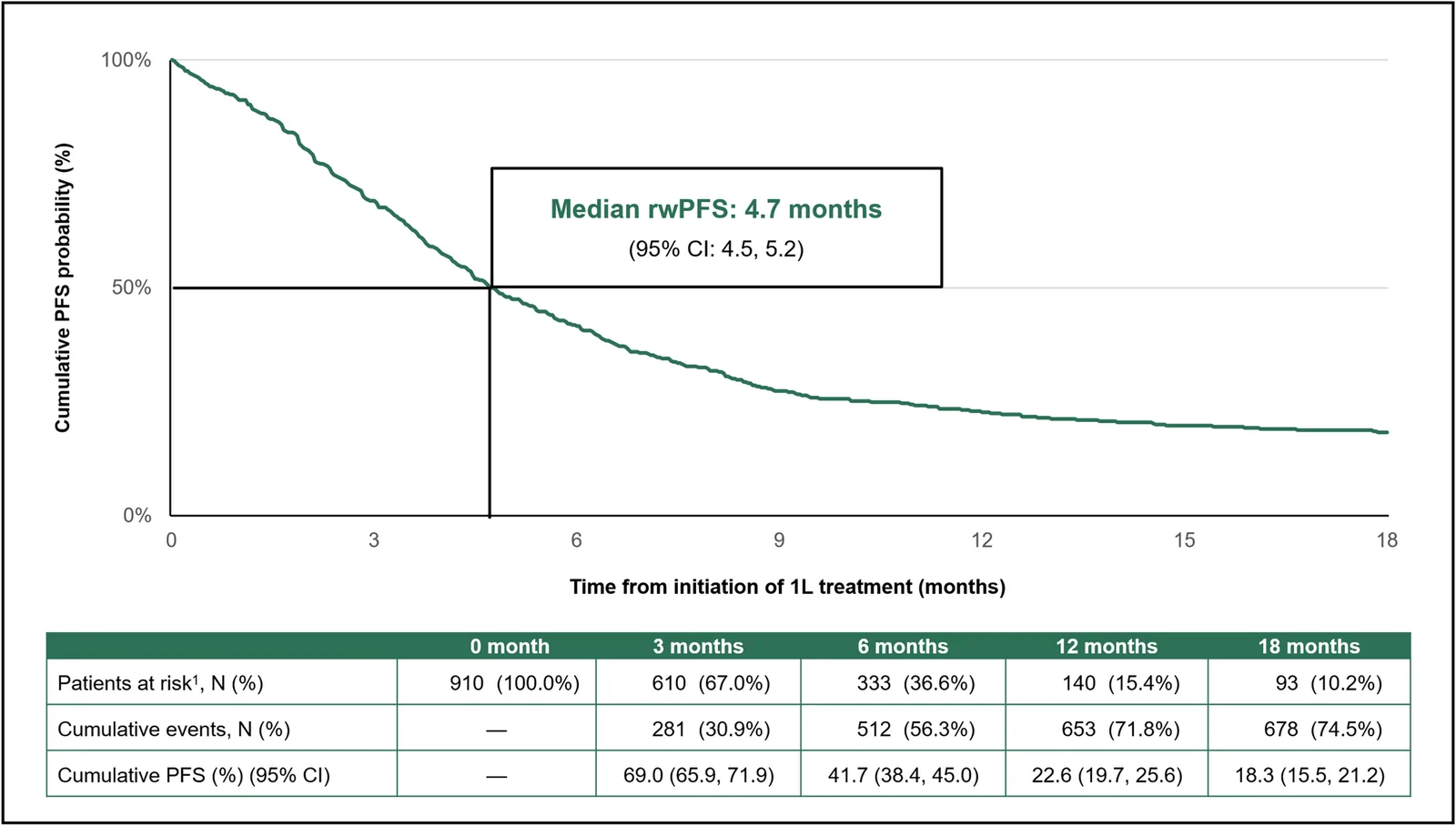
rwPFS from initiation of 1L treatment for PD-L1-negative lr/mTNBC. Abbreviations: 1L: first line; CI: confidence interval; lr/mTNBC: locally recurrent inoperable or metastatic triple-negative breast cancer; PD-L1: Programmed Death-Ligand 1; rwPFS: real-world progression-free survival. Note: 1. Kaplan-Meier analyses were assessed among patients who initiated their 1L regimen for lr/mTNBC ≥ 6 months prior to the end of data availability

A total of 910 patients had sufficient follow-up to assess rwPFS. Of them, 703 (77.3%) had an indicator of progression, with a median rwPFS of 4.7 (95% CI: 4.5, 5.2) months (Fig. 4). rwPFS rates at 3, 6, and 12 months were 69.0%, 41.7%, and 22.6%, respectively. The breakdown of the first real-world indicators of progression is shown in Supplemental Fig. 3.

## Discussion

This retrospective cohort study used a large US administrative claims database to describe real-world treatment patterns and clinical outcomes among patients with PD-L1-negative lr/mTNBC. Our study demonstrated substantial heterogeneity in treatments received, with 329 distinct treatment sequences observed in this population, and the most commonly used 1L regimen (i.e., AC-based therapy) being discordant with current National Comprehensive Cancer Clinical Practice Guidelines in Oncology (NCCN Guidelines^®^) [17]. Clinical outcomes following initiation of 1L treatment were poor, with a median rwOS of approximately one year and a short time of approximately 5 months to progression. Taken together, these findings suggest that the 1L systemic therapies currently used in routine practice for patients with PD-L1-negative lr/mTNBC have limited effectiveness and the need for more effective treatment options to improve clinical outcomes.

Preferred regimens for the treatment of PD-L1-negative lr/mTNBC in 1L, as recommended by the NCCN Guidelines ^®^, available throughout the study period included single-agent systemic chemotherapy (e.g., capecitabine, paclitaxel) [17]. However, treatment patterns observed in this study were not aligned with guideline-recommended approaches, with just 12.7% and 10.4% of patients receiving capecitabine or paclitaxel in 1L, respectively, while the most frequent regimen category observed was AC-based therapy (28.1%), which is recommended as useful in certain circumstances by the NCCN Guidelines [17]. Reported use of AC-based regimens in lr/mTNBC varies across prior studies, with some analyses describing higher proportions (e.g., Bhave et al.: 43.9%), while the majority reported lower levels (e.g., Skinner et al.: 11.1%; Jaber Chehayeb et al.: 10.2%) [10, 18, 19]. Variation in the use of AC-based therapy may reflect differences in patient characteristics, particularly the proportion of recurrent inoperable versus *de novo* metastatic cases, which can influence regimen selection. In the present study, approximately two thirds of patients had *de novo* mTNBC, a substantially higher proportion than that reported in many clinical trials, such as the ASCENT-03 and TROPION-Breast02 trials, where *de novo* metastatic cases typically represent closer to one third of the study population [14, 15]. Notably, among patients receiving AC-based therapy in this study, 94.1% had *de novo* metastatic disease. Rocque et al. (2018) suggested that patients with *de novo* mBC may be more likely to receive aggressive combination chemotherapy at metastatic diagnosis as these patients have not previously received systemic therapy in the early-stage or adjuvant setting [20].

Additionally, patients in this study were treated with several different 1L regimens, an observation also reported in previous real-world studies of treatment patterns in patients with lr/mTNBC [10, 21, 22]. The heterogeneity in treatment approaches observed in real-world clinical practice likely reflects the challenges in treating this patient population, particularly because patients with PD-L1-negative lr/mTNBC have no approved targeted treatment options in the 1L setting.

Furthermore, a substantial proportion of patients (40.5%) either discontinued all treatment after 1L or died while receiving their 1L regimen and therefore did not proceed to subsequent therapy. Several factors may contribute to this high proportion, including rapid clinical deterioration, limited treatment options, or preference to transition to supportive care in the setting of aggressive disease. Fewer than half (44.5%) were observed to receive 2L therapy, and only 16.0% of all patients received a 3L regimen. High attrition rates have also been observed in previous real-world studies of patients with lr/mTNBC, which have reported 51.0% and 57.0% of patients receiving 2L therapy, and 26.0% and 33.6% of patients receiving 3L therapy [21, 22]. The higher proportions observed in those studies may partly reflect the inclusion of PD-L1-positive patients, who are eligible for targeted immunotherapies and may therefore be more likely to continue to later LOTs.

Taken together with the high attrition across LOTs, clinical outcomes in this cohort were poor, with rwOS and rwPFS estimates highlighting the limited effectiveness of available treatments. The median rwOS (12.2 months) and median rwPFS (4.7 months) observed in this study align with previous real-world studies of patients with lr/mTNBC treated with various systemic therapies, which have reported median rwOS values ranging from 11.3 to 14.6 months, and rwPFS from 4.2 to 6.5 months [10, 21, 22]. In these prior studies, which evaluated patients treated between 2010 and 2022 and included predominantly PD-L1-negative populations (with a subset of PD-L1-positive patients), most patients received guideline-concordant therapy, with only 11.1–16.8% receiving AC-based regimens. These cohorts also had a lower proportion of *de novo* metastatic disease (21.0–29.9%) and, in most cases, smaller sample sizes. These findings suggest that although AC-based therapies are more intensive, their use may not confer substantially improved survival in real-world practice. Further, clinical outcomes in this population were poorer than those reported for other mBC subtypes. Patients with non-TNBC mBC typically have higher median rwOS, ranging from 42.0 to 60.1 months, and survival has improved over the years, potentially reflecting the increasing availability of targeted therapies for these subgroups [21, 23, 24]. These differences underscore the need for additional treatment options capable of improving outcomes for patients with this more aggressive subtype. Additionally, findings were consistent in a sensitivity analysis restricted to patients treated after the introduction of PD-L1 targeted agents, supporting the robustness of the results despite changes in the treatment landscape.

Results from this study are subject to several limitations. First, disease stage and biomarker status (e.g., ER, PR, HER2, and PD-L1) are not directly available in the claims database used in this study, thus the identification of the study sample relied on algorithms developed with medical expert input. In particular, biomarker-defined subgroups were approximated using treatment-based proxies, which may result in misclassification as the absence of targeted therapy does not definitively indicate biomarker negativity and may reflect factors such as treatment availability or approval timing. However, results from a sensitivity analysis restricted to patients with index date on or after March 2019 (approval of atezolizumab in this setting) were consistent with the primary findings, supporting the robustness of the results. Similarly, because clinical notes were not available, the descriptions of LOTs, rwPFS, and the imputed death dates used to supplement information in rwOS analysis were derived from algorithms developed with input from medical expert. Although this is a limitation, the claims-based algorithms used in this study reflect established approaches that are commonly employed in the literature, and results were found to be similar to studies using clinical notes [25]. Second, this study assessed outcomes among patients eligible for systemic therapy. As such, outcomes may vary in the broader lr/mTNBC population, including those who are not candidates for systemic therapy. Third, this study is subject to common limitations inherent to retrospective analyses using claims data, including the risk of missing or miscoded data as well as inaccurate insurance claims. Lastly, given the rapidly changing treatment landscape for TNBC, future studies with sufficient follow-up are needed to assess the impact of these medications on real-world clinical outcomes.

## Conclusion

In conclusion, this retrospective study provides contemporary real-world evidence on treatment patterns and clinical outcomes from 1L initiation in patients with PD-L1-negative lr/mTNBC in the US. Patients were treated with highly heterogeneous and often guideline-discordant 1L regimens, experienced short duration of therapy, and showed substantial attrition between treatment lines. Clinical outcomes remain poor, with rapid disease progression after initiation of 1L therapy and rwOS of approximately one year despite the use of intensive combination chemotherapy in many patients. Together, these results underscore the substantial unmet need in PD-L1-negative lr/mTNBC and highlight the critical need for new, effective, and tolerable treatment options for this patient population with unfavorable prognosis.

## Data Availability

The data that support the findings of this study are available from Komodo. Restrictions apply to the availability of these data, which were used under license for this study.

## Declarations

### Previous presentations

Part of the material in this manuscript was presented as poster presentations at ESMO Congress 2025, held October 17th – 21st , 2025, in Berlin, Germany and SABCS 2025, held December 9th – 12th , 2025, in San Antonio, Texas, USA.
